# Characterizing human random-sequence generation in competitive and non-competitive environments using Lempel–Ziv complexity

**DOI:** 10.1038/s41598-021-99967-6

**Published:** 2021-10-19

**Authors:** Alice Wong, Garance Merholz, Uri Maoz

**Affiliations:** 1 Department of Psychology, University of California Los Angeles, Los Angeles, CA USA; 2 Institute for Interdisciplinary Brain and Behavioral Sciences, Chapman University, Irvine, CA USA; 3 Department of Psychology, Crean College of Health and Behavioral Sciences, Chapman University, One University Drive, Orange, CA 92866 USA; 4 Computational and Data Sciences, Schmid College of Science and Technology, Chapman University, Orange, CA USA; 5 Anderson School of Management, University of California Los Angeles, Los Angeles, CA USA; 6 Biology and Bioengineering, California Institute of Technology, Pasadena, CA USA; 7 Université de Paris, CNRS, Integrative Neuroscience and Cognition Center, 75006 Paris, France

**Keywords:** Psychology, Human behaviour

## Abstract

The human ability for random-sequence generation (RSG) is limited but improves in a competitive game environment with feedback. However, it remains unclear how random people can be during games and whether RSG during games can improve when explicitly informing people that they must be as random as possible to win the game. Nor is it known whether any such improvement in RSG transfers outside the game environment. To investigate this, we designed a pre/post intervention paradigm around a Rock-Paper-Scissors game followed by a questionnaire. During the game, we manipulated participants’ level of awareness of the computer’s strategy; they were either (a) not informed of the computer’s algorithm or (b) explicitly informed that the computer used patterns in their choice history against them, so they must be maximally random to win. Using a compressibility metric of randomness, our results demonstrate that human RSG can reach levels statistically indistinguishable from computer pseudo-random generators in a competitive-game setting. However, our results also suggest that human RSG cannot be further improved by explicitly informing participants that they need to be random to win. In addition, the higher RSG in the game setting does not transfer outside the game environment. Furthermore, we found that the underrepresentation of long repetitions of the same entry in the series explains up to 29% of the variability in human RSG, and we discuss what might make up the variance left unexplained.

## Introduction

It has been debated whether true randomness exists in nature; some have even further claimed that randomness cannot be clearly defined^1,2^. Nevertheless, characteristics that have been associated with random series include equiprobability of terms and sequential independence^1^. Formally, for a binary series ${\left({s}_{i}\right)}_{i=1}^{n}\in {\left\{\mathrm{0,1}\right\}}^{n},$ equiprobability means that for any $b\in \left\{\mathrm{0,1}\right\}$, $p\left({s}_{i}=b\right)=0.5$—i.e., each item has a 50% chance of being a 0 or a 1. More generally, equiprobability means that for any $b\in \left\{\mathrm{0,1},\dots ,m-1\right\}, p\left({s}_{i}=b\right)=\frac{1}{m}$; or, for $m$ items, each has a $\frac{1}{m}$ probability of taking each of the $m$ values. Sequential independence—also “memorilessness” or negative recency^3^—means $p\left({s}_{i}=b|{s}_{i-1},\dots ,{s}_{1}\right)=p\left({s}_{i}=b\right)$. In other words, sequential independence means that any entry in a series is not influenced by the entries before it (or after it).

Randomness—particularly Random Series Generation (RSG)—is often used in neuroscience and psychology to increase cognitive load—e.g., as a distractor task from a main task^4–6^. However, research has also been carried out specifically on the behavioral, cognitive, and neural underpinnings of RSG in humans. It has thus been shown that, when instructed to carry out RSG, humans can generate series that are empirically equiprobable when the number of items to randomize is small—e.g., 0’s and 1’s or rock, paper, scissors—though less so for a larger number of items—e.g., the digits from 0 to 9^7^. However, regardless of the number of items, they fail to make those series sequentially independent^1,8^ (see Tune^9^ and Wagenaar^10^ for reviews of the classic literature on this topic).

To better understand sequential independence in human RSG, let us first define a *run* in a series as a sequence of the same entry [e.g., the series (0, 0, 0, 0, 1, 1, 0) is made up of 3 runs: the four consecutive 0’s, the two consecutive 1’s, and the final 0]. Bearing this definition of a run in mind, it has been shown that humans tend to produce systematically biased series, switching too often between entries, and thus underrepresenting long runs in their generated series^8,11^. More generally, it has been shown that humans tend to underrepresent the previous item in the series, yet overrepresent items that are 2 and 3 back, regardless of whether they were instructed to vocalize or write the items down^12^.

That said, there is evidence that, after a lot of training, humans are able to produce series that are more sequentially independent; however, that result should be taken with a grain of salt: first those series were not empirically equiprobable (over the digits from 0 to 9), second, this was only shown on a single participant, who was also the experimenter and thus especially committed^7^. It has been further demonstrated that, with detailed instructions and when participants can see their generated series, human RSG becomes more sequentially independent and thus more random^8,11^. In particular, in a competitive environment with feedback—e.g., a matching-pennies game—humans exhibit higher sequential independence while apparently maintaining empirical equiprobability^2,11^. In addition, expert-level players in games where randomness provides a competitive advantage have been shown to be more random than novice players during the game, though this randomness did not extend to post-game non-competitive RSG^13^. However, while human randomness has been shown to be context dependent, it has not (to the best of our knowledge) been directly compared to non-human objective benchmarks—e.g., to pseudorandom series generation (pseudo-RSG) by computer algorithms.

In the discussion of randomness above, we paid special attention to sequential independence and equiprobability. However, various other characteristics of random sequences have been proposed. In particular, the National Institute for Standards and Technology in the US has provided guidance for random sequences (SP800-22) that includes 15 different tests of randomness^14^ (e.g., complexity tests, spectral tests, compressibility). And it is difficult to say which, among this multitude of criteria, are the most important characteristics of randomness. This is perhaps not surprising given the lack of consensus on how to even define randomness. We therefore decided to use a measure of compressibility as our measure of randomness (reminiscent of Maurer’s Universal Statistical Test^14^) that we term the Lempel–Ziv Complexity (LZC), which is based on a compression algorithm developed by Ziv and Lempel^15^ (see “Methods”: “Data analyses” for more detail on this algorithm).

The extent of human randomness has implications in various branches of neuroscience, psychology, and beyond. In the neuroscience of volition, for example, the ability to act randomly relates to the focus of that field on arbitrary choice (a prototypical example is reaching for one carton of milk on a supermarket shelf lined with identical ones—same brand, fat content, etc.; a typical experiment in that field may instruct participants to randomly raise their left or right hand at the go signal on every trial)^16^. This focus on arbitrary (or random) choice and action follows from the assumption that such arbitrary action, devoid of any reasons or valuation of alternatives, reflects the most distilled type of free choice^17–20^. And, while this view has been criticized^21–24^, it remains central in the field. To judge the extent to which participants in such experiments acted randomly, as they were instructed, it is important to know how random humans can be. So, in this respect, a person’s maximal randomness sets an upper limit on the arbitrariness of their actions in experiments like the above.

Here we aim to integrate three strands of research across multiple disciplines. One pertains to how human behavior is shaped by competitive contexts, also studied in economics and game theory^2,11^. The second compares human RSG to computer pseudo-RSG—i.e., measuring human random behavior in relation to a comparable random (but non-human) process. And the third contextualizes the implications human RSG has for understanding cognitive processes within multiple fields of psychology related to volition, decision-making, psychopathology, and the underlying brain structures that support these processes. More specifically, we aim to answer the following questions. (1) Can human RSG in a game situation be improved when informing people that they must be as random as possible to win the game? (2) Is human RSG in a game environment similarly random to the pseudo-RSG commonly used in modern programming languages? (3) Is the improved RSG exhibited in a game environment transferrable to post-game RSG? (4) To what extent are changes in RSG ability related to changes in the length of generated runs?

## Methods

### Participants

153 undergraduate students [112 female, age 20.7 ± 2.2 years (mean ± SD)] were recruited through the Psychology Department participant pool at the University of California, Los Angeles (UCLA). The study was approved by UCLA’s North General Institutional Review Board (NGIRB; IRB#15-001094). All procedures were performed in accordance with the Declaration of Helsinki. Written informed consent was obtained from all participants, and all individuals received course credit for participation. Participants were recruited in 3 batches. After the first batch, we established how many more participants we would need to oversample from each condition during the 2nd and 3rd batches to end up with roughly the same number of subjects in the Unaware and Aware conditions.

We carried out the power analysis to have enough power to test, using a mixed-effect ANOVA—whether being more random during the game would translate into being more random in the post-game RSG and whether being or not being aware of the need to be random would affect the degree of randomness (see below). Based on previous literature, we assumed a small effect size of 0.2. To obtain a power of 0.8 at a significance level of 0.05, we calculated that we would need to collect data from n = 102 participants (assuming no correlation in a repeated-measures ANOVA). We opted to recruit 50% more participants to be conservative. Our results then showed that the effect size in our data was 0.17, slightly less than 0.2. A post-hoc power analysis suggests that n = 141 participants are needed to obtain a power of 0.8 with a significance level of 0.05 at this effect size. As we were left with n = 142 participants after exclusion (see below), our analysis suggests that our results are not underpowered.

### Experimental design and procedure

**Figure 1:**
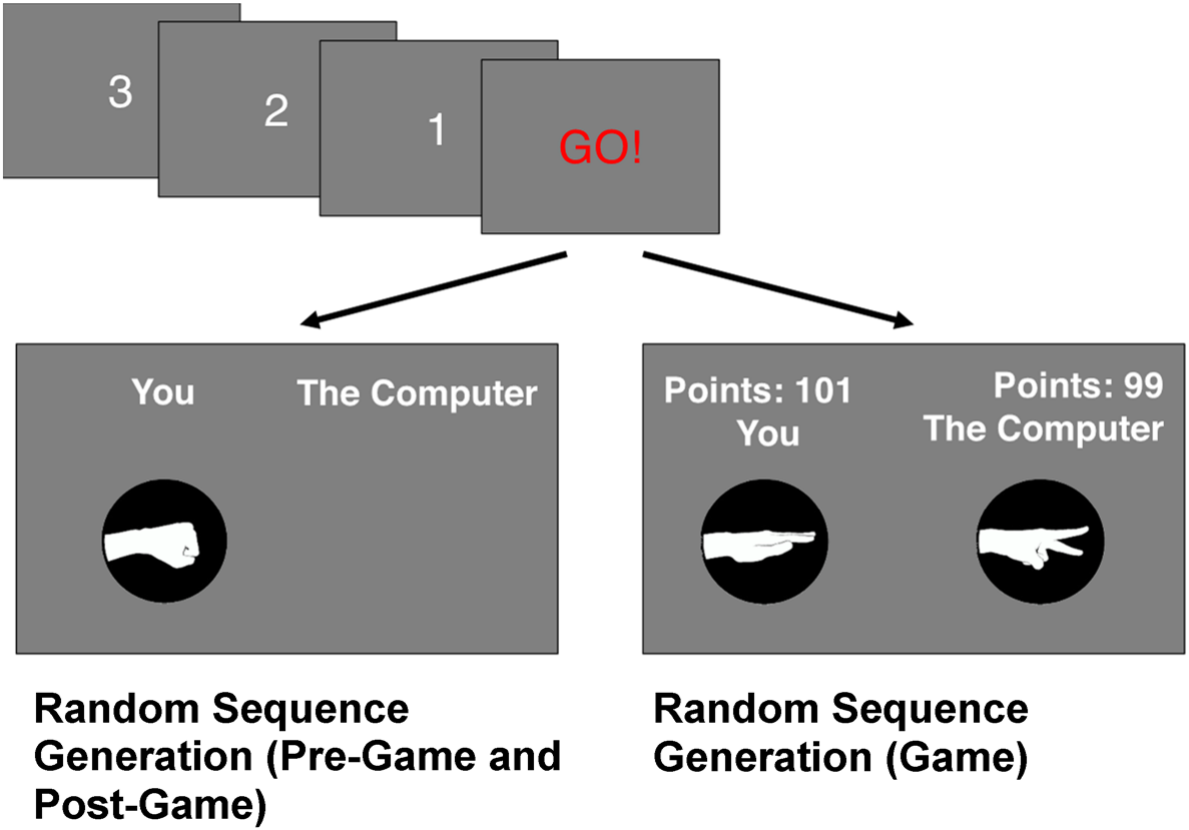
The progress of each trial in the experiment. Following the countdown, participants were required to press the appropriate key for rock, paper, or scissors within 500 ms of the onset of the Go signal; otherwise, the trial was forfeit. Their selection was then presented on the screen. In Game trials (on the right), their selection was accompanied by the computer’s selection and by the Game score.

The experiment was designed as a $3\times 1$ between-participant study—with the conditions being (1) *Unaware*, (2) *Aware*, and (3) *Control*. Each experimental session was divided into 3 parts, with 200 trials per part, separated into 2 blocks of 100 trials. All participants completed all 3 parts of the experiment. In each trial, participants selected Rock (R), Paper (P), or Scissors (S) by pressing the <G> , <H> , or <B> keys on the keyboard, respectively. In the first—*Pre-Game*—part of the experiment, participants were instructed to endogenously generate a sequence of R–P–S that was as random as possible. For the second—*Game*—part of the experiment, participants were told they would be playing R–P–S with the computer as their opponent, and their goal was to win as many points as possible. During the game, the computer used a specific strategy to either predict or not the participant’s next move, depending on the experimental condition (see below). In the third and final—*Post-Game—*part of the experiment, the Pre-Game instructions were repeated. An experimental trial is depicted in Fig. 1.

In the Game part only, the participant and the computer started with 100 points each. The winner was the player with more points at the end of the Game. Each player gained or lost 1 point for every trial they won or lost, respectively. Ties left the score unchanged. Participants were further randomly assigned into one of 3 conditions during the Game. In all conditions they were instructed to do their best to win the Game. However, participants in the Unaware and Control conditions were told only that—i.e., they knew nothing about the computer’s strategy during the Game. Those in the Aware condition were explicitly informed that the computer would try to predict their moves and it was stressed that to win they must be as random as possible (Table 1).

**Table 1:** Within- and between-participants division of the experiment.

Pre-game	Game	Post-game
Random sequence generation	*Condition 1, Unaware* Computer exploits patterns in participant’s choice history against the participant. Participant is not informed of computer’s strategy	Random sequence generation
	*Condition 2, Aware* Computer exploits patterns in participant’s choice history against the participant. Participant is explicitly informed of computer’s strategy and told that to win they must be random	
	*Condition 3, Control* Computer follows simple pattern continuously with 85% probability on each trial and acts randomly with 15% probability. Participant is not informed of computer’s strategy	

Each participant carried out all three parts of the experiment (Pre-game, Game, and Post-game). Different participant groups were randomized into conditions 1–3 only in the Game part of the experiment.

In the Unaware and Aware conditions, the computer sought out behavioral patterns in the participant’s choice history (e.g., R–R–P–P–S–S–…), their win/lose/tie patterns (e.g., win-stay-lose-switch) and used any patterns it could find against them (for details on the Matlab algorithm used see^22,25^). Briefly, the algorithm predicted the most likely next play (R, P, or S) given the previous plays and their outcomes (win/loss/tie). The prediction was then based on the conditional probabilities of all the possible next moves given the history of moves and their outcomes. The next play that the algorithm output was the possibility that followed the most likely pattern, or in other words, the one that was least likely by chance (attached Matlab file for the implementation of the computer’s playing algorithm).

Given the computer’s playing algorithm, the participant’s best strategy was to be as random as possible, minimizing the computer’s ability to find patterns in their choice history. In the Control condition, the computer followed the pattern R–P–S–R–P–S–… cyclically with 85% probability on each trial, with a remaining 15% chance of choosing R, P, or S (with equiprobability) on each trial. At the end of the experiment, the participants were then given a post-experiment questionnaire asking them about the task, and their performance in the game (see more about the questionnaire below).

### Participant- and trial-inclusion criteria

#### Data exclusion and errors

**Figure 2:**
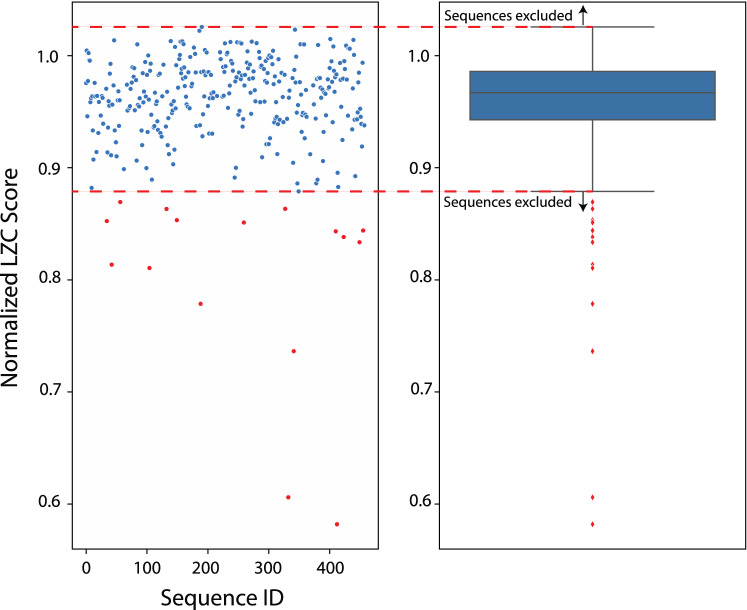
Visualization of exclusion criteria. Scatter (left) and box (right) plots showing the LZC scores of all the sequences in the Unaware and Aware conditions. Sequences more than 1.5*IQR away from the median were identified as outliers and shown in red. See Supplementary Table 1 for a list of excluded sequences and for more on sequence IDs.

We identified outlier sequences using a typical 1.5*IQR (IQR = inter-quartile range) exclusion criterion—i.e., any sample more than 1.5 away from the median (Fig. 2). The 16 sequences with LZC scores that were above or below the median ± 1.5*IQR from any part of the experiment (not including the Control condition) were identified as outliers (Supplementary Table 1 contains all the excluded sequences). We did not include sequences from the Control condition in our exclusion calculation as sequences generated during the Game in the Control condition were, by design, intended not to be random and hence to elicit low-LZC scoring sequences. This was because of the repetitive patterns of the series generated by the computer player. This meant that most participants also generated repetitive pattern, which they needed to do to win the Game in the control condition. Note that due to the results we obtained, we ended up removing the Control condition from the main manuscript, relegating it to the Supplementary instead.

Of the 111 total participants in the Unaware and Aware conditions, 12 (10.8%) generated the 16 sequences that met the exclusion criteria above. So, these 12 participants were thus excluded from further analysis according to the criterion outlined above (10 were excluded from the Unaware condition, 2 from the Aware condition). Another way to exclude participants is by 1.5*IQR distance from the mean—separately for the Unaware and Aware conditions. It should be noted that this method results in largely the same participants being excluded (the only difference is that Participant #57 is then not excluded from the Unaware condition; see Supplementary Table 1).

We expected more exclusions from the Unaware condition because of the experimental setup. It was rather difficult to beat the computer. And participants in the Unaware condition had to implicitly learn how to win in the Game part, while those in the Aware condition were explicitly told how to beat the computer. We anticipated an imbalance in the exclusion numbers between the Unaware and Aware conditions, so we deliberately assigned more participants to the Unaware condition during participant randomization. We therefore ended up with 51, 48, and 42 participants in the Unaware, Aware, and Control conditions, respectively, or 141 participants overall.

On top of participant exclusion, there were also two types of errors that could occur on each trial. (1) The participant did not press a key within 500 ms of the Go signal, and (2) the participant pressed a key other than <G> , <H> , or <B> . Both errors resulted in a forfeited trial. Further—in the Game part—a point was awarded to the computer in such cases without a loss of a point for the participant. Both errors were rate. The first occurred on 3.1 ± 2.6% of all trials and the second on 0.5 ± 0.8% of all trials (mean ± STD across participants). Those trials were removed from further analysis.

#### Post-experiment questionnaire

Of the 141 participants, who were left after exclusions, 117 (83.0%) answered the post-experiment questionnaire. (This is because the questionnaire was introduced only after the first batch of participants completed the experiment.) The questionnaire consisted of the following 7 questions. In each of them we asked the participants to rate how much they agreed with the following 7 statements, each on a Likert scale of 1 to 7 (1 = strongly disagree, 7 = strongly agree):

1. Whether they used a strategy against the computer.
2. Whether they felt that the computer was predicting their moves.
3. Whether they managed to create very random sequences in part 1 (before the game) and part 3 (after the game).
4. Whether the sequence they created in part 3 was more random than the one they created in part 1.
5. Whether they won most of the trials during the game.
6. Whether they tied on most trials during the game.
7. Whether they lost most of the trials during the game.

### Data analyses

#### Measure of randomness

To quantify the randomness of a given sequence, we used a normalized Lempel–Ziv Complexity (LZC) score, which has been only sparsely used in previous research^26^.

This measure of randomness has the added benefit of avoiding narrow definitions relying only on sequential independence and equiprobability. The LZC score is based on the LZ78 algorithm, which is a variant of a dictionary-based, lossless, compression algorithm developed by Ziv and Lempel^15,27^. The LZC score is obtained by comparing the ratios of the length of a sequence before and after compression with the LZ78 algorithm. The algorithm starts with a pre-initialized dictionary of single characters, and then reads in the next character of the original sequence until the resulting substring no longer matches any known substring in the dictionary. Then, this new substring is appended to the dictionary with a new code consisting of the index of the last known substring plus the latest new character. The algorithm thus achieves compression by replacing repeated substrings in the original, uncompressed sequences with a shorter code (see attached Python file for the specific implementation used in this analysis).

We then normalized the LZC score by generating 1000 pseudorandom sequences of the same length as the human generated sequence, and divided the human generated sequence score by the mean LZC score of the pseudo-randomly generated sequences^28^. This was to remove the effect of small variations in sequence length on the raw LZC score, due to the removal of error trials, as a shorter sequence may naturally be less complex than a longer sequence due to its length. Thus, a normalized LZC score closer to 1 indicates that a sequence is more complex, and thus random, whereas a score closer to 0 indicates that a sequence is less random. As an example, take a sequence [3 2 1 2 1 2 1 2 2 3], which is of length 10. After compressing it with the LZ78 algorithm, the sequence is stored as [3 2 1 257 269 2 3] (see Ziv and Lempel^15^ for details). The length of this compressed sequence is therefore 7 (generally speaking, the LZ78 algorithm tends to result in more dramatic compression for longer sequences). To normalize it, we take 1000 pseudorandom sequences of length 10, so perhaps ${x}_{1}=\left[1 2 1 1 3 1 2 2 2 1\right]$, ${x}_{2}=\left[ 2 1 3 1 1 2 1 2 1 2\right]$, …, ${x}_{1000}$ = $\left[3 3 2 2 3 3 1 2 3 3\right]$. Then the normalized LZC score is $\frac{7}{mean(length\left(lz78\left({x}_{i}\right))\right)}$.

It is worth noting that we did not use any measures of randomness that directly rely on sequential dependence because, in the Game part for the Unaware and Aware conditions, the computer algorithm relied on this measure to play against the participant. Thus, using a similar pattern-matching algorithm to test for randomness could amount to double-dipping, as participants were already evaluated against this measure by feedback from the algorithm in the Game.

#### Permutation tests

To compare the randomness scores of the subjects to those generated by the computer pseudorandom number generator, we had to first understand the range of the pseudorandom normalized LZC scores. We therefore generated 1000 pseudo-random sequences of R–P–S of length 192 (the length of the average human sequence).

**Figure 3:**
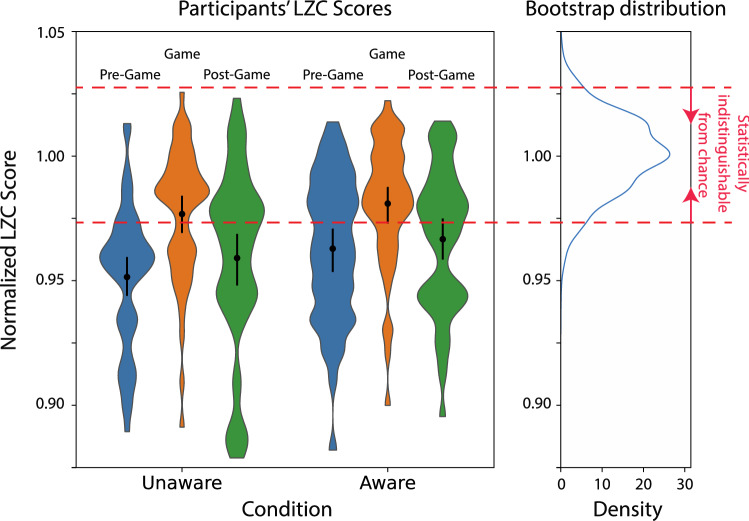
LZC scores of human generated sequences. Violin plots of LZC scores by experimental part and condition are shown with the mean and 95% confidence intervals superimposed in black are shown on the left. The empirical distribution of 1000 bootstrapped pseudo-random sequences is given on the right. The horizontal, solid red lines—on the left and right—indicate the mean LZC score at the 2.5th and 97.5th percentile (bottom and top, respectively) of 1000 bootstrapped, computer-generated pseudo-random sequences. Values between these lines are statistically indistinguishable from the computer-generated pseudo-random sequence distribution. (See Supplementary Fig. 2 for all LZC scores, including the Control condition).

Then, for each pseudorandom sequence separately, we ran the identical code that we used on the human data to compute their randomness scores. This gave us 1000 randomness scores to which we could compare the randomness score that we computed on the human data (see Fig. 3). The pseudo-random sequences were generated by Python’s pseudo-random-number generators (using the Mersenne twister algorithm). We then took 5% of the randomness scores as the critical threshold ($\alpha$ level) for LZC scores (see below) to designate significant deviations from chance level. The bootstrapped 2.5th to 97.5th percentile of the normalized LZC score distribution was [0.973, 1.027] for a sequence length of 192. Thus, for a random sequence as random as Python’s random-number generator, a sequence’s normalized LZC score can be expected to fall between 0.973 and 1.027 with a 5% a level in a two-tailed test. The theoretical upper limit for an LZC score is 1. However, because the sequences were all generated empirically, the upper range of the normalized LZC score can exceed 1 in our calculations, if the numerator sequence’s LZC score happened to be larger than the average mean of 1000 sequences other sequences.

#### Statistical testing

We used null-hypothesis statistical testing (NHST) as well as Bayesian hypothesis testing for our statistical testing. Generally speaking, NHST enables the researcher only to reject H_0_ given a low-enough p value (that p value indicating the probability that we would have found our data or more extreme data if H_0_ was correct), then stating that there is a statistically significant difference between some conditions (as per H_1_). In contrast, Bayesian analyses—and the Bayes factor (BF_10_) in particular—deal with the amount of evidence favoring H_1_ (i.e., that the conditions are different) versus H_0_ (i.e., that the conditions are the same). Hence, Bayesian analysis can conclude that the evidence supports H_1_, supports H_0_, or is inconclusive. Following Rouder^29^, we took a BF_10_ odds ratio between 3 and 10 as reflecting “some evidence,” between 10 and 30 as “strong evidence,” and greater than 30 as “very strong evidence” for H_1_ over H_0_. The reciprocals (i.e., one over 3, 10, and 30) would be respectively taken as corresponding evidence for H_0_ over H_1_. Finally, 1/3 < BF_10_ < 3 is taken as inconclusive evidence for either H_1_ or H_o_. All our Bayesian analyses were conducted using the JASP software package^30–32^.

Bayesian analysis requires us to make assumptions about the prior distribution of the parameter in which we are interested, which affects the BF_10_ we get. However, we found no literature on priors to use in Bayesian statistical tests for RSG. We therefore opted to use JASP’s default prior: the Cauchy distribution with a scale/parameter of 0.707. First, selecting a prior in advance guards against cherry picking a prior that supports gaining evidence for one’s favored hypothesis. Second, we often use Bayesian inference here to seek evidence for H_0_. And using other, wider priors results in lower BF_10_—indicating stronger evidence in favor of H0. So, results favoring H_0_ based on this default prior are thus relatively robust to change of priors.

#### Explanatory variable: average run length

We also computed an explanatory statistic called the run-length—the mean length of a run in a series. For instance, the run-length of the sequence R–R–R–P–P–S–P–P is the mean of the lengths of all the runs—3, 2, 1, 2—which is 2. The run-length is related to the randomness of a sequence in that the longer the run-length of a sequence is, the fewer runs there will be for a sequence of fixed length. We bootstrapped this measure, similarly to the randomness scores above, to obtain an empirical distribution of mean run-lengths. The 2.5th to 97.5th percentile range was [1.363, 1.658]. For a perfectly sequentially independent sequence of 3 items, the ideal run-length is 1.5.

## Results

### R–P–S game results (manipulation check)

Three of our four research questions relate directly to the degree of randomness that our participants could achieve during the Game part in the Unaware and Aware conditions. Yet the participants were to be driven to relatively high randomness by striving to beat a computer algorithm that used patterns in their behavior against them. In contrast, the Control condition was developed to make subjects play the game while not acting randomly (see “Methods”). We therefore wanted to test that our participants struggled against the computer in the Unaware and Aware conditions and did relatively well in the Control condition.

We therefore analyzed the final game scores in the 3 conditions: Unaware 75.78 ± 17.63, Aware 78.83 ± 16.06, Control 140.10 ± 51.05 (mean ± STD). This suggest that our manipulation worked well. Participants found it difficult to beat the computer in both the Aware and Unaware, where the winning strategy required them to be random. On the other hand, participants in the Control condition, where the winning strategy was to almost always follow the simple, cyclic P-S-R pattern, found it easy to win (Supplementary Fig. 1).

Overall, the experimental manipulation created a large difference (in terms of effect size^33^) between conditions. After exclusions (see “Methods”), 3 out of 51 (5.88%), 4 out of 48 (8.33%), and 31 of 42 (73.81%) participants beat the computer in the Unaware, Aware, and Control conditions, respectively. Per our design, participants were much more likely to lose to the computer in the Unaware and Aware conditions, than under the Control condition using NHST; but there were no statistically significant differences between the Unaware and Aware conditions. In other words, instructing the participants that they needed to be as random as possible to beat the computer in the Aware condition resulted in no statistically significant increase in the number of participants who were able to beat the computer in comparison to the Unaware condition (ANOVA with Welch correction for homogeneity, F(2, 79.52) = 30.43, p < 0.001, ${\eta }_{p}^{2}=0.46$; post-hoc t-test comparison with Bonferroni correction for Unaware vs Aware, t(97) = − 0.49, p = 1.0, Cohen’s d = − 0.18, 95% CI [− 17.92, 11.83]. The Bonferroni is a conservative correction for multiple comparisons that is most commonly used, and thus enables easy comparison with other literature.) The consistent differences (i.e., the significant p-value in the main effect of the ANOVA) were due to the differences between the Control condition and the experimental conditions (post-hoc t-test Unaware vs Control: t(91) = − 9.89, p < 0.001, Cohen’s d = − 1.75, 95% CI [− 79.72, − 48.90]; Aware vs Control: t(88) = − 9.28, p < 0.001, Cohen’s d = − 1.67, 95% CI [− 76.89, − 45.63]).

Using a Bayesian analysis, we corroborated the above and found very strong evidence that a larger number of participants won during the Control condition in comparison to the other two experimental conditions. Importantly, this analysis also provided some evidence that the Unaware and Aware conditions were the same. (Bayesian ANOVA specifying a multivariate Cauchy prior on the effects; the model with condition as an effect best represented the data over the null model with no effects, BF_10_ = 2.12 × 10^16^. Post-hoc Bayesian t-tests for Unaware vs Control had a BF_10_ = 1.09 × 10^10^, and for Aware vs Control, BF_10_ = 8.12 × 10^8^. Post-hoc comparison corrected for multiple testing between the Unaware and Aware conditions: BF_10_ = 0.30).

### Measuring and comparing randomness

We measured the randomness of the series that our subjects generated using LZC scores (see “Methods”). The distributions of the LZC scores for each condition and part of the experiment are shown as violin plots in Fig. 3 (see also Supplementary Table 2 for full descriptive statistics).

### Can human RSG in a game situation be improved when informing people that they must be as random as possible to win the game?

As per the research question, from the onset we were interested in whether the degree of randomness was similar or different between the Game parts of the Aware and Unaware conditions (Fig. 3, left). We therefore carried out a planned comparison directly between the normalized LZC scores in the Game part of the Aware and Unaware groups. An NHST comparison between the two groups indicated that there was no significant difference between the randomness scores (unpaired t-test t(97) = − 0.78, p = 0.43, Cohen’s d = − 0.16).

We then wanted to further investigate whether there was evidence that the randomness in the Aware and Unaware conditions was the same. We thus ran a Bayesian t-test between the normalized LZC scores in the Aware and Unaware conditions and found some evidence that our participants were equally random in the two conditions (BF_10_ = 0.28).

### Is human RSG in a game environment similarly random to the pseudo-RSG commonly used in modern programming languages?

We wanted to test how similar human RSG is to the pseudo-RSG that is generated in modern programming languages. We therefore used a permutation test to compare Python’s RSG to the RSG of our participants (see “Methods”). We found that—in the Game part only, and during the Aware and Unaware conditions only—the mean normalized LZC scores were above the 2.5 percentile of computer-generated bootstrapped distribution (Fig. 3, right; note that, as this is a permutation test, just the mean of those scores, and not their mean ± 95% CI, needs to be above the 2.5 percentile of the bootstrapped distribution). So, the LZC scores in the Game part during the Aware and Unaware conditions are statistically indistinguishable from those of Python’s random-number generator.

### Is the improved RSG exhibited in a game environment transferrable to a post-game environment?

Following previous literature, we expected participants to display higher randomness during the Game part in comparison to the Pre-Game and Post-Game parts in both the Aware and Unaware conditions. However, we wanted to know whether subjects were able to maintain the higher randomness that they achieved during the Game part in the Post-Game RSG. As expected from previous literature, we found significantly higher normalized LZC scores during the Game parts in both conditions. But it did not seem that subjects were able to maintain this higher level of randomness in the Post-Game part of the experiment in both conditions (Fig. 3). Participants’ level of randomness in the Pre- and Post-Game parts were consistently smaller than during the Game part but not consistently different from each other (repeated measures ANOVAs with a between-subjects factor (condition: Unaware, Aware) and within-subjects factor (Experiment part: Pre-Game, Game, Post-Game) to compare the LZC scores across conditions and across experiment parts, (F(2,194) = 19.81, p < 0.001, ${\eta }_{p}^{2}=0.17$). Post-hoc paired t-tests with Bonferroni correction for multiple comparison revealed that participants were significantly more random during the Game than in the Pre-Game or Post-Game. (Pre-Game vs. Game: t(97) = − 6.1, p < 0.001, Cohen’s d = − 0.61, 95% CI [− 0.03, − 0.01]; Game vs. Post-game: t(97) = 4.47, p < 0.001, Cohen’s d = 0.45, 95% CI [0.01, 0.03]). The Pre-Game level of randomness, however, was not significantly lower than the Post-Game (Pre-Game vs. Post-Game: t(97) = − 1.60, p = 0.11, Cohen’s d = − 0.16, 95% CI [− 0.014, 0.003]).

A Bayesian analysis corroborated the NHST results, providing very strong evidence for higher randomness during the Game part compared to the other parts in both the Aware and Unaware conditions (Bayesian repeated-measures ANOVA model (specifying a multivariate Cauchy prior on the effects), with the experiment part only as a factor versus the null model with no effects using JASP: BF_10_ = 1.29 × 10^6^, which is very strong evidence for this model over the null model. There was also very strong evidence in favor of the model with the experiment part and condition both as factors over the null model (BF_10_ = 1.06 × 10^6^) as well as for the model with both factors (experiment part and condition) including an interaction term between the factors (BF_10_ = 1.04 × 10^5^). However, the Bayesian analysis did not provide conclusive evidence that the Aware and Unaware conditions were the same or different (post-hoc comparisons for Unaware vs. Aware: BF_10_ = 1.19). In sum, we found very strong evidence that participants were more random during the Game part than the Pre- and Post-Game conditions but no evidence that the Pre- and Post-Game parts were different (though we found no evidence that they were similar either).

In addition, there was no consistent difference between the levels of randomness in the Aware and Unaware conditions (overall post-hoc paired t-test with Bonferroni correction between the Aware and Unaware conditions showed no significant difference (t(79) = − 1.82, p = 0.07, Cohen’s d = − 0.18, 95% CI [− 0.02, 0.006]). A Bayesian analysis did not find conclusive evidence for consistent similarity between the two experimental parts, though the evidence was trending toward the same randomness in both parts (post-hoc Bayesian t-test corrected for multiple comparisons between the Pre-Game and Post-Game parts: BF_10_ = 0.39).

On top of the aggregate analysis above, we also tested LZC scores on a subject-by-subject basis. In the Unaware condition, 35 of the 51 participants, 68.63%, were more random (according to their LZC scores) during the Game than during both Pre- and Post-Game, which is significantly more than expected by chance (binomial test p < 0.001; chance level is 25%). Similarly, in the Aware condition, 25 of the 48 participants, 52.08%, were more random during the Game than in both Pre- and Post-Game, again more than expected by chance (binomial test p < 0.001). Hence, both the average and individual LZC scores suggest a pattern of participants being more random in the Game compared to the Pre-Game and Post-Game for the Aware and Unaware conditions.

To further test whether participants had different Pre- and Post-Game LZC scores, we ran a subject-by-subject analysis. In the Unaware condition, we found that 32 out of 51 participants had a lower LZC score in the Pre-Game than in the Post-Game (binomial test, p = 0.16), which was not significantly more than expected by chance. In the Aware condition, 26 out of 48 participants had a lower LZC score in the Pre-Game than in the Post-Game (binomial test, p = 0.89), which was not significantly more than expected by chance. Therefore, we find that the pattern of individual LZC scores also provided no evidence for a difference between Pre-Game and Post-Game randomness.

As the LZC did not remain higher after the Game into the Post-Game RSG, the Control condition that we developed to specifically control for that became less important. We therefore relegated the figures that included the Control condition (Supplementary Figs. 2 and 3) and the statistical analysis of the information within those figures to the Supplementary Material.

### To what extent are changes in RSG ability related to changes in the length of generated runs?

**Figure 4:**
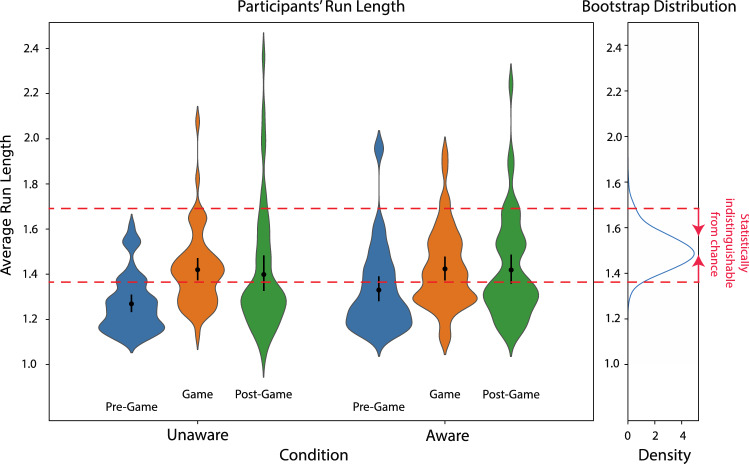
Average run-length scores of human generated sequences. Average run-length scores by experimental part and condition are indicated below each violin plot and in black inside each violin plot with 95% CI. The horizontal red line indicates the mean run-length of the 2.5th percentile of 1000 bootstrapped pseudo-random sequences. (See Supplementary Fig. 3 for all LZC scores, including the Control condition).

It is known that humans underrepresent long runs when carrying out RSG. Investigating further why participants’ LZC scores varied in the above pattern, we therefore looked at the length of the runs that the participants were creating, comparing the average run-lengths of participants’ sequences across conditions and Game parts (Fig. 4).

The pattern of the run-length results mirrored that of the LZC scores only to a certain extent (see Supplementary Table 3 for full descriptive statistics). This time, participants produced significantly longer average run lengths during the Game and Post-Game parts in comparison to the Pre-Game (Repeated-measures ANOVA with Greenhouse–Geisser correction for sphericity comparing the Unaware and Aware conditions, average run-lengths across the experiment parts were significantly different (F(1.8,174) = 20.84, p < 0.001, ${\eta }_{p}^{2}=0.18$; post-hoc paired t-tests with Bonferroni correction for multiple comparisons: Pre-Game vs. Game: t(97) = − 5.87, p < 0.001, Cohen’s d = − 0.59, 95% CI [− 0.17, − 0.07]; Game vs. Post-Game: t(97) = 0.62, p = 1, Cohen’s d = 0.06, 95% CI [− 0.04, 0.06]; Pre-Game vs. Post-Game: t(97) = − 5.26, p < 0.001, Cohen’s d = − 0.53, 95% CI [− 0.16, − 0.06]). We also found no significant effect of condition (overall post-hoc paired t-tests with Bonferroni correction: Unaware vs. Aware, t(97) = − 0.89, p = 0.38, Cohen’s d = − 0.09, 95% CI [− 0.09, 0.04]).

Bayesian analysis corroborated the NHST results, providing very strong evidence for longer run-length during the Game and Post-Game parts in comparison to the Pre-Game, in both the Aware and Unaware conditions (Bayesian repeated-measures ANOVA model—specifying a multivariate Cauchy prior on the effects—determined that the data were best represented by a model that included experiment part only, as a factor (BF_10_ = 2.2 × 10^6^), compared to the null model. Post hoc Bayesian t-tests (controlled for multiplicity): Pre-Game vs Game BF_10_ = 1.47 × 10^7^, Pre-Game vs Post-Game BF_10_ = 31,444.42).

In contrast to the above evidence for differences in run length between some experiment parts, we found some evidence for the same average run length between the Game and Post-Game parts (post hoc Bayesian t test: BF_10_ = 0.13). We also found some evidence that there was no difference in average run length between the Unaware and Aware conditions (post hoc Bayesian t test: BF_10_ = 0.25).

This trend also held for individual participants. In the Unaware condition, 32 of the 51 participants, 62.75%, had longer runs during the Game than during both Pre- and Post-Game, which is significantly more than expected by chance (binomial test p < 0.001). For the Aware condition, 23 of the 48 participants, 47.92%, had longer runs during the game than during both Pre-Game and Post-Game, which is once more significantly more than expected by chance (binomial test p = 0.002). Note that these proportions of participants with longer runs during the Game in comparison to the other 2 experiment parts were not significantly different between the Unaware and Aware conditions (${\chi }^{2}$ test: ${\chi }^{2}$=0.63, p = 0.43).

We wanted to understand the extent to which the variance in randomness might be explained by the run length. We therefore regressed each sequence’s average runs-length onto its corresponding LZC score. We found a significant correlation between the two overall and for each experiment part separately. Overall, using simple OLS regressions, the run length explained almost a fifth of the variance in randomness across all experiment parts and conditions combined (R^2^ = 0.19, p < 0.001; see Supplementary Fig. 4 for details). It also explained more than a quarter of the variance in the Pre-Game (R^2^ = 0.27, p < 0.001; see Supplementary Fig. 5 for details) almost three tenths of the variance in the Game (R^2^ = 0.29, p < 0.001; see Supplementary Fig. 6 for details) and a tenth of the variance in the Post-Game (R^2^ = 0.1, p < 0.001; see Supplementary Fig. 7 for details).

### Questionnaire answers

As part of the post-experiment questionnaire, 117 of our participants reported whether they used a strategy against the computer in the game (n = 44 for unaware, n = 34 for aware n = 39 for control). We thus tested whether there was a difference between their responses in the Aware and Unaware subject groups. Subjects’ median rating was 4 out of 7 (IQR = 2.75–5) for Unaware, and 4 out of 7 (IQR = 1.25–5) for Aware, and 6 out of 7 (IQR = 4.5–7) for Control.

The mean ratings for the Unaware was 3.71 ± 1.67, 3.53 ± 1.89 for Aware, and 5.28 ± 1.97, for Control (mean ± STD). Hence, the Aware and Unaware conditions were both reliably different from the control but not from each other (ANOVA F(2, 114) = 10.44, p < 0.001, ${\eta }_{p}^{2}=0.16$. Post-hoc independent t-tests: Unaware vs Aware t(76) = 0.41, p = 1; Unaware vs Control t(681) = − 3.87, p < 0.001; Aware vs Control t(71) = − 4.03, p < 0.001.

As the Likert items give ordinal data, we also tested group differences with the Kruskal–Wallis Test. We found an overall significant difference of condition (H(2) = 19.54, p < 0.001). Dunn’s Post-hoc comparisons with Bonferroni adjustment for multiple comparisons showed that Unaware vs. Aware was not significantly different from each other (p = 1), while Unaware vs. Control and Aware vs. Control were significantly different (p < 0.001).

In a similar manner, we tested whether subjects’ perceptions of being predicted by the computer differed between the Aware and Unaware condition. Subjects’ median rating was 5 (IQR = 2.75–6) for Unaware, 5 ((IQR = 4–6) for Aware, and 4 (IQR = 2–5.5) for Control. The mean ratings were 4.36 ± 2 (Unaware, mean ± STD), 4.65 ± 1.82 (Aware), and 3.67 ± 2.16 (Control), which were not significantly different between conditions (ANOVA F(2, 114) = 2.37, p = 0.098, ${\eta }_{p}^{2}$ = 0.04. Kruskal–Wallis Test H(2) = 4.25, p = 0.12).

We also tested whether participants’ perception of their improved ability to generate randomness after the Game correlated with their actual improvement in randomness as measured by their LZC score. The correlation between their answer to questionnaire question 4 (whether the sequence they created Post-Game was more random than the one they created Pre-Game) and the difference in LZC score between the Post-Game and Pre-Game sequences was small and not statistically significant (Pearson’s R = − 0.11, p = 0.26. Spearman’s rho = − 0.11, p = 0.24). Similarly, we tested the correlation between the LZC score difference and ratings of question 3 (whether they managed to create very random sequences in Pre-Game and Post-Game). Again, the correlation was very small and not significant (Pearson’s R = − 0.02, p = 0.82. Spearman’s rho = − 0.06, p = 0.52).

### Gender

Because our sample was imbalanced with regards to gender (after exclusions, we were left with 101 female vs. 40 male participants over all conditions), we conducted an analysis to see whether our results differed by gender (see Supplementary Table 4 for full descriptive statistics). To verify this, we ran a repeated measures ANOVA with two between-subjects factor (condition: unaware, Aware; gender: male, female) and a within-subjects factor (experiment part: pre-Game, Game, Post-Game). We did not find significant differences in LZC scores by gender (F(1,95) = 0.275, p = 0.60, ${\eta }_{p}^{2}=0.003$.) and even found some evidence that there is no difference between males and females with a Bayesian analysis (BF_10_ = 0.21). So, we conclude that the gender imbalance is unlikely to significantly bias our results.

## Discussion

Multiple researchers (e.g., Wagenaar^10^, Mookherjee and Sopher^34^, Nickerson and Butler^8^, Rappaport^2,11^) have previously demonstrated that humans are unable to generate highly random sequences. That said, previous literature has also demonstrated that humans are able to be more random in competitive situations with feedback^2,11^. Building on that, we set out to examine four research questions. First, we tested the extent to which human RSG in a competitive game situation depended on whether the subjects were specifically informed that they must be as random as possible to win the game. We found some evidence that the degree of randomness when our participants were aware versus unaware that they must be as random as possible to win was the same. So, in other words, simply informing subjects that they must be as random as possible did not make them any more (or less) random.

Other factors could have naturally contributed to this similar degree of randomness between the Unaware and Aware groups. For example, Hyman and Jenkin^35^ demonstrated for a similar task that subjects’ motivation to succeed and their belief in their ability to succeed in the task contribute to their performance. Our participants may have lacked incentives to be particularly motivated to win in the game context, as their performance was not related to any compensation, or even to an overall competition among the participants for points, unlike other studies^11^. Participants in the Aware and Unaware groups might have also believed that the computer’s prediction algorithm works well and that they were unlikely to be able to beat it.

Interestingly, the post-experiment questionnaire suggests subjects reported similar levels of confidence that they used a strategy against the computer in the Aware and Unaware conditions. Hence, being made aware that they needed to be random to beat the computer did not make subjects more likely to use a conscious strategy against the computer than when they were unaware that they needed to be random. Similarly, we found no difference in subjects’ perceptions of how much the computer predicted them between the Aware and Unaware conditions. In both cases they felt slightly predicted. So, again, being made aware that the computer was predicting them did not make subjects feel more predicted by the computer. Furthermore, the post-experiment questionnaire confirmed that participants’ perception of the randomness of their sequences had very little correlation with the actual randomness of those sequences. Together, these results support previous findings that RSG seems decorrelated from consciousness^13^ and suggest that it may be an automatic, involuntary process.

Studies in animal models have shown that a competitive setting triggers strategic counterprediction, rather than stochastic behavior, even when acting as random as possible maximizes reward^36–38^. Perhaps participants cannot help but try to predict the computer's next actions, before attempting to be as unpredictable as possible themselves, even when they have been informed that the latter is the optimal strategy. This automatic counterprediction can also explain why Morra players did not exhibit better randomization in the game setting^13^, where counterprediction is just as important as stochastic unpredictability to win.

Intriguingly, the neural underpinnings of RSG in a competitive setting differ between animal and human models: in animals, the nodes which are recruited during strategic rule-building, most notably the anterior cingulate cortex (ACC), become decorrelated during stochastic behavior^36^. In humans, on the contrary, ACC activation covaries positively with randomization performance^39^, suggesting that humans use a different mechanism to produce random sequences. Moreover, it seems that a widespread, inter-connected recruitment of various regions is needed to produce more random sequences^40^, much like the organization thought to support conscious access to stimuli^41^. This seems opposed to the various findings of decorrelation between RSG and consciousness but may be explained by a need to fully recruit one's conscious attention onto the task itself; this may then not leave any resources for becoming conscious of the specific mechanisms used to produce a sequence that is as random as possible. It may also be what makes the difference between humans and rodents in terms of RSG neural mechanisms.

Our second research question was how random can people be in a competitive game environment? We found, as far as we know for the first time, that human RSG in this environment was on average statistically indistinguishable from pseudo-RSG algorithms commonly used in modern programming languages. This was the case at least when participants had to be as random as possible to win (i.e., in the Unaware and Aware conditions, but not in the Control condition). So, combined with the first research question, our results suggest that human RSG in a game cannot be further improved, beyond its high level, by explicitly telling participants that they need to be random to win.

That said, the extent to which computer pseudo-RSG algorithms are random is debatable. (Python’s default pseudo-random generator relies on the Mersenne twister algorithm—the same goes for Matlab, Stata, Ruby, R, Julia, Scilab, SageMath, etc.—which passes the Diehard tests of randomness and the Small Crush battery of the newer TestU01 collection, but fails on the more extensive Crush and Big Crush battery of the TestU01 collection^42–44^; (TestU01^45^ is a software library used to benchmark and test the performance of uniform random number generators. It consists of 3 predefined batteries of statistical tests named “Small Crush” (containing 10 tests, e.g., Marsaglia’s birthday spacings test, Knuth’s simple poker test) “Crush” (96 tests), and “Big Crush” (106 tests) in increasing order of stringency and runtime.). Our results are nevertheless interesting and set a higher benchmark for the ability of humans to be random than was previously known.

We previously hypothesized that the similarity between the levels of randomness in the Unaware and Aware conditions during this Game part stemmed from subject’s lack of motivation. However, the high level of randomness that we found during the Game part—statistically indistinguishable from modern, algorithmic RSG—casts serious doubt on that hypothesis.

Third, we were wondering whether the improved RSG exhibited in a competitive game environment would transfer to a post-game environment—i.e., are people more random after the game than they were before the game? We did not find evidence for such a transfer of human RSG ability when looking at the LZC score. Put differently, we found no evidence that subjects were able to maintain the higher degree of randomness that they exhibited during the Game also Post-Game. We also asked them explicitly about their RSG ability Pre- and Post-Game. And we found that, at the group level at least, participants were generally not able to perceive when they were more or less random Post-Game. Thus, it appears that participants were able to transfer neither the implicit nor the explicit randomness that they exhibited during the Game to their RSG after the game. One potential explanation for this effect may be that being random is a costly, effortful task, which requires a lot of cognitive resources, and can thus only be achieved under the higher motivational state when playing a game.

Last, Nickerson^1,8^ and colleagues have shown that humans underrepresent long runs of short sequences during RSG—i.e., that humans over alternate between items in the series—and this is one reason for the limited human RSG ability. We therefore wanted to test whether changes in RSG ability were related to changes in the length of generated runs. We found that the run-length explained between 10 and 29% of the variance in participants’ degree of randomness. Therefore, average run-length by itself is apparently far from sufficient to characterize randomness as measured by the LZC.

What is more, while Post-Game LZC scores dropped back to Pre-Game levels, the average run-length did not drop down from the Game level in the Post-Game. In addition, run-length explained only 1/10 of the variance in the Post-Game RSG, which is much lower than the amount of variance that it explained during the Pre-Game and Game parts. This suggests that participants managed to learn to make longer runs in the Post-Game—as long as in the Game—but their sequences nevertheless ended up being roughly as random as those in the Pre-Game. This might be because the run-length measure tracks only the repetition of unigrams (i.e., single entries in the series), whereas the LZC is sensitive to repetition of $n$-grams for any $n\ge 1$. Thus, previous research which characterized human RSG with measures of randomness less sensitive to $n$-grams than the LZC may have missed post-game transfer effects relating to a return to higher-order pattern frequency (i.e., repetition of larger $n$-grams post-game).

Following the above, it would be interesting to investigate what factors make up the remaining 70–90% of the variability in human randomness that was left unexplained by run length. For example, giving instructions in a more intuitive manner, or using a different method of eliciting random sequences may reveal different results. Wagenaar^10^ hypothesized a difference between verbal instruction and visual display of potential entries in the random sequence which could affect random sequence generation. Ayton et al.^46^ further suggests that some of the observed non-randomness may come from instructional bias from the experiment, by giving explicitly prohibited examples of unlikely or unacceptable sequences (e.g., telling participants that the sequence “ABC” or meaningful words are unlikely when drawing letters randomly). Though Beach and Swensson^47^ showed that specific instructions related to the gambler’s fallacy—e.g., telling subjects to ignore run dependency—do not help lengthen participants’ run lengths. They suggest that the method of eliciting random sequences may instead be more important. This is in line with the results of Peterson and Ulehla^48^, who had participants take part in a probability learning task and found that they did better when they had to learn the probabilities in a series formed by them throwing dice than by them picking stacked cards (even though the stacked cards were ordered according to another participant’s dice throws).

Regardless of the above, it remains unclear what factors make our participants more random in the competitive game environment compared to the post-game. There are at least two differences between the Game and Post-Game trials: the competitive nature and the feedback in the former. A future study could test whether post-game randomness changes after a non-competitive game with constant feedback on one’s level of randomness (e.g., via a continuously updated runs-test score), potentially teasing apart the effect of feedback from competition. Competition and feedback could also increase motivation, which might further explain the difference in randomness (e.g., through the mechanism of attention). So, it would be good to test whether introducing a monetary reward above a certain randomness threshold, for example, could improve randomness (Hyman and Jenkin^35^ suggest it may). In addition, explicitly informing participants that randomness during a game is higher than during pre-game might make them realize that they do better during the game and facilitate transferring their increased randomness to the post-game environment.

Recent results suggest that individual RSG ability could potentially be like a fingerprint—a unique biomarker of cognition^49–51^. RSG ability also appears to vary amongst different psychopathological populations^52–54^. It appears, from our results, among others, that humans cannot easily and consciously modulate their randomness at will. This might explain the randomness-as-a-biomarker phenomenon, and it might also provide support for the use of RSG as a clinical diagnostic tool. For example, if RSG is driven by momentary neural noise, these noise patterns might be individual specific, or they could be modulated by some diseases. If so, it would be interesting to test whether these individual RSG patterns generalize from self-paced to competitive game environments and vice versa.

It may further be of interest to go beyond our behavioral results toward a neural model of randomness. In this respect, our results may relate to the “Network-Modulation Model” for randomness proposed by Jahanshahi and colleagues. The model suggests that the superior temporal cortex (STC) is involved in stereotyped responses (such as ordered counting) and that suppression of these habitual responses is necessary for RSG. Further, according to the model, this suppression is achieved via the left dorsolateral prefrontal cortex (DLPFC), which exerts an inhibitory influence on the STC. The model was constructed based on evidence from positron emission tomography, transcranial magnetic stimulation, and lesion studies^55–57^. This model more recently gained support from electroencephalography studies as well^58,59^. However, if DLPFC activity can be upregulated to exert enough inhibitory influence on the STC during Game trials, why was this ability not transferred to the Post-Game trials of our experiment? Note that our task instructions informed subjects that the Post-Game RSG would follow the Game part. One possibility is that something in the game environment triggered the DLPFC’s inhibitory influence on the STC to create more-random sequences; however, even though participants were instructed to be as random as possible in the Post-Game RSG, and thus likely attempted to do so consciously and voluntarily, the DLPFC did not exert the same level of inhibition on the STC in the post-game part. If DLPFC activity could be brought under more voluntary control—e.g., via neurofeedback^60^—would up- or down-regulating left DLPFC activity then increase or decrease participants’ level of randomness, respectively? It would be interesting to test these speculations empirically as part of a future neuroscientific study. In particular, a neuroimaging version of our task could test whether the higher degree of randomness during a game correlates with stronger inhibition of the STC by the DLPFC, or whether other circuits also alter their activity, thereby increasing DLPFC activity or decreasing STC activity, in accordance with the Network-Modulation Model.

On top of the more general relation to neuroscience, our results more specifically relate to the neuroscience of volition. For example, our finding that RSG in a competitive environment might be as random as a computer’s pseudo-RSG mean that the upper limit on the arbitrariness of human action could be higher than previously realized—at least in a competitive game environment. In addition, previous work has demonstrated the existence of biases that may affect decisions, especially when the decision alternatives are of similar value^22^. Interestingly, those biases were found in the DLPFC, which has also been implicated in RSG. It would therefore be interesting to investigate the extent to which biases in decision making and RSG are supported by the same neural mechanisms, which may involve the DLPFC.

Several potential limitations of our study should be mentioned. One stems from its gender imbalance. We found no evidence for gender differences in RSG generation in the literature. And we found evidence that there are no differences in RSG ability between males and females in our dataset. Nevertheless, our participants were 73% female and this roughly 3:1 gender imbalance might still have some effect on our results. Future research with a large-enough dataset that is roughly gender balanced might dispel this concern.

Another limitation of our study was our inability to account for the great majority of the variance in the LZC measure using other factors that we measured; less than 1/3 of the LZC variance was explained by the run-length scores. What is more, the relation between the LZC score and run length may be more complex than simply linear. Future research could track other factors, beyond run length, that would enable a better characterization of the variance in the LZC score. Finally, our data were collected on the Rock–Paper–Scissors game and RSG. It is unknown to what degree our results would generalize to games with more or less choice items, as well as perhaps to more complex games.

## Supplementary Information

Supplementary Information.
