# Supplementary material for: Characterizing human random-sequence generation in competitive and non-competitive environments using Lempel–Ziv complexity
Source: Sci Rep. 2021 Oct 19;11:20662. doi: 10.1038/s41598-021-99967-6 (PMC8526708; doi:10.1038/s41598-021-99967-6)
Supplement: Supplementary file 1 — Supplementary Information. [file 41598_2021_99967_MOESM1_ESM.docx]

**Characterizing human random-sequence generation in competitive and non-competitive environments using Lempel-Ziv complexity—Supplementary material**

Alice Wong^1,2,4^, Garance Merholz^1,7^, Uri Maoz^1,2,3,5,6*^

**Supplementary Figure 1.** Visualization of final game score by condition. Final scores above the dashed line indicates that the participant won over the computer. Sequences are sorted by the final-score difference between human and computer.

**Supplementary Figure 2**. LZC scores for the Unaware, Aware, and Control conditions.

For the Control condition, subjects’ scores versus those of the computer (Supplementary Fig. 1), suggest that participants realized that the computer was generally using a consistent strategy of R-P-S, with rare deviations (see Methods). Hence, we found that—as per our design—subjects were less random on average during the game compared to the pre-game and post-game (repeated measures ANOVA (Greenhouse-Geisser correction), looking at the control condition, LZC scores across the experiment parts significantly different (F(2, 123) = 11.87, p < 0.001, $\eta_{p}^{2}=0.16$)). Post-hoc paired t-tests with Bonferroni correction for multiple comparisons showed that participants were significantly less random during the game than during the pre-game and post-game, with the pre-game and post-game not significantly different from each other (Pre-game vs. Game: t(40) = 4.09, p < 0.001, Cohen’s d = 0.77, 95% CI [0.02, 0.1]; Game vs. Post-game: t(40) = -4.34 p<0.001, Cohen’s d = -0.85, 95% CI [-0.10, -0.03]; Pre-Game vs. Post-game: t(40) = -0.25, p=1, Cohen’s d = -0.09, 95% CI [-0.04, 0.01]). As for subject-by-subject results, 8 of 42 were less random during the game than during both pre- and post-game, which is not more than expected by chance (chance level of 0.25%, binomial test p=0.284).

**Supplementary Figure 3.** Average Run-length for for the Unaware, Aware, and Control conditions.

In the control condition, no significant differences in run-length was found among the 3 parts of the task (repeated-measures ANOVA with Greenhouse-Geisser correction for sphericity, F(2, 82) = 0.47, p=0.63, $\eta_{p}^{2}=0.01$). The analysis of individual participants did not result in any reliable differences either: 17 of 42 participants had longer runs during the game than during both pre- and post-game, 40.5%, and this was not more than expected by chance (binomial test p = 0.07).

**Supplementary Figure 4.** OLS regression of average run-length on LZC score for all experiment parts and conditions combined


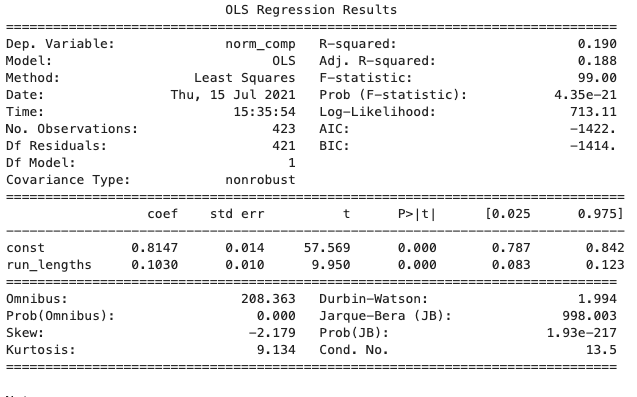


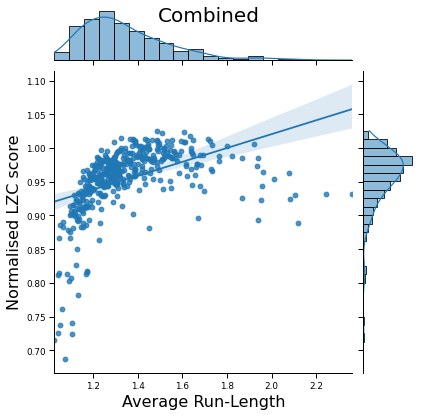


**Supplementary Figure 5.** OLS regression of average run-length on LZC score for the Pre-Game part of the experiment.


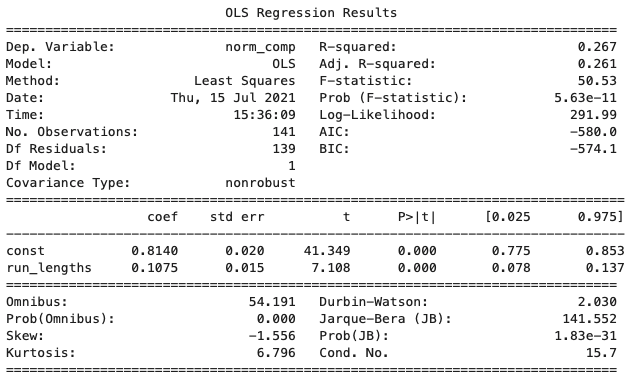


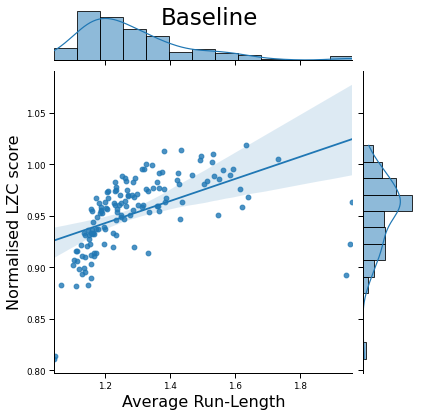


**Supplementary Figure 6.** OLS regression of average run-length on LZC score for the Game part of the experiment.

**
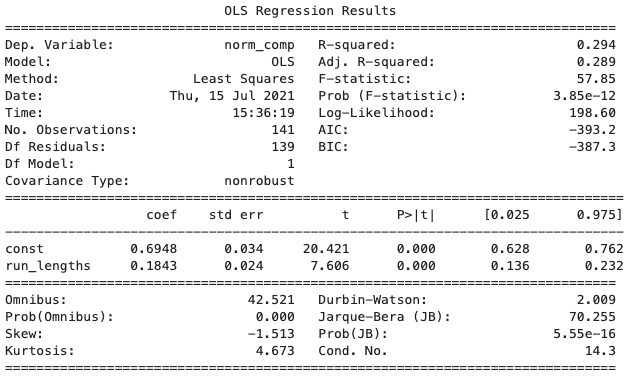

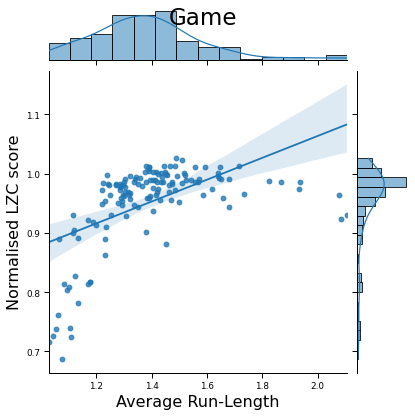
**

**Supplementary Figure 7.** OLS regression of average run-length on LZC score for the Post-Game part of the experiment.


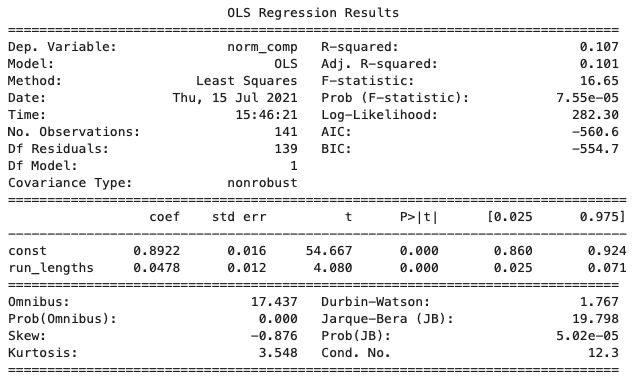

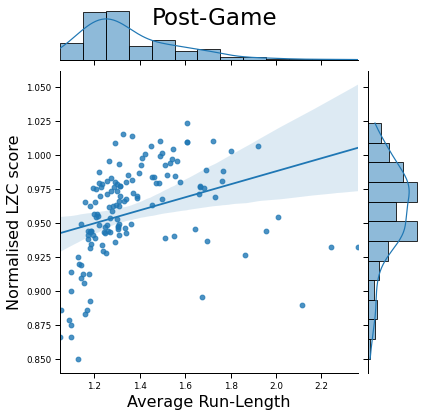


**Supplementary Table 1.** All the sequences identified as outliers. 1 = Rock, 2 = Paper, 3 = Scissors. Note that 4 participants (#36, 105, 107, and 150) were excluded because of more than one series.

| **Participant ID,**  **Condition type, Experiment part, (Sequence ID)** | **Sequence** | **Sequence**  **length** |
| --- | --- | --- |
| 22,  Unaware  Post-Game,  (327) | 11322132312312312123123123123111213123123122332221123213121321321321331321321223312113121232132132113213123121321321231233123112312312312312331231231231231233123123312311213221312313123131321322311 | 198 |
| 27,  Unaware  Post-Game,  (332) | 3211321212322212222222222321222222222222213222221322222222222222222222222222222222222222232111221111111112222222222222222222222222222222223132323113122222222222222222222222222222222222 | 185 |
| 35,  Unaware  Pre-Game,  (34) | 3231231321312321231213213211323132313212312312312312312313121321321321231231232123123121213321321231231312232213231231213231231231232131221313212131123121321313212321231123131231223131231231321232 | 197 |
| 36,  Unaware  Game,  (188) | 2211112111111111111111111111111131111111212112222333111111111111111112113211111121111111123111111111111111211211211111112111111111221111121111121311233131313131313132222223312333333323232323232111111 | 200 |
| 36,  Unaware  Post-Game,  (341) | 12333333311111122222221111112223331111222223333331112222221122333311222222211333112211111111112222233311122223333311112222222222222222223333333331122222222222222221113333333333333333333333332 | 192 |
| 43,  Unaware  Pre-Game,  (42) | 12233311322232323333222222233333233311111123212233333333321233312331322333331333333333333333133333333222221111333331333122222222222222222222233232113323111111111111131213222222333231333333 | 189 |
| 57,  Unaware  Pre-Game,  (56) | 123132132312313212312312313212123123131231232323131321321323132123132132132321312131313212123132123231233123121313132123123123221231333231233212121123123123231123213213232313232132332312312113112 | 196 |
| 105,  Aware  Pre-Game,  (104) | 13231321321321323123123123121213123123132132131232123131323132132132132131231231231213231323132131231231232312312123313332132312132132323123121321231213213121231231231231231231213212132123132132132 | 198 |
| 105,  Aware  Post-Game,  (410) | 12312123123123123312321321231213213212312312132123121321323132132312132132122213132132132131213212132132132312332312321232113232112212131212131212312332332132123123312312321313322132132132132 | 192 |
| 107,  Unaware  Game,  (259) | 123333332122222222222332222112111332212312232323123123112113222222112112112121211111211313211111222112112123123121111313111111111333322113233233111133122231111111111111111111111111111111111 | 190 |
| 107,  Unaware  Post-Game,  (412) | 11122233311122233311122211111111111111111111111122222222222222222222222222222222311111111111111111111111111112333333333333331111111111112222222233333111111122222222222222222222211111111111111111 | 195 |
| 118,  Unaware  Post-Game,  (423) | 221212111231221112123333333332333233333332333223222121231231112312121311132333333333333222232233333122211112222212222222222233333333322333333322222222222212121112333322333333333 | 178 |
| 133,  Aware  Pre-Game,  (132) | 1232231312313213321321321332121332123113221333232321231321321321321321231233231313212321231323213132131321132132312132132123132123123213213213223112113233221232313212132123231123213223123312332 | 194 |
| 144,  Unaware  Post-Game,  (449) | 112123122231123123123123123123123123122311111112111121113132131112111111111111111111121211111111211111111111111112112121212222212111211211111122121111111111222213213213132321111111112311123131232312 | 199 |
| 150,  Unaware  Pre-Game,  (149) | 123231212123132312132321231231231231322121213322312312312312312312312312123123131213213212332131233332131213321232312323112313213213231132323132121223213213231323123132131323123132132132132132 | 193 |
| 150,  Unaware  Post-Game,  (455) | 21231321313212313231322313212312313113212313221321232213212313131322112132122332123132123322121321312323132312132321321322132123113231321321232132132132131231321321322132131231321321321321321 | 192 |

**Supplementary Table 2. Descriptive statistics for LZC scores in all conditions and game parts.** Each participant carried out all three parts of the experiment (pre-game, game, and post-game).

| **Condition** | **Experiment part** | **Mean** | **STD** |
| --- | --- | --- | --- |
| Unaware | Pre-Game | 0.951 | 0.028 |
| Unaware | Game | 0.977 | 0.027 |
| Unaware | Post-Game | 0.959 | 0.037 |
| Aware | Pre-Game | 0.963 | 0.029 |
| Aware | Game | 0.981 | 0.026 |
| Aware | Post-Game | 0.967 | 0.029 |
| Control | Pre-Game | 0.943 | 0.047 |
| Control | Game | 0.885 | 0.095 |
| Control | Post-Game | 0.946 | 0.036 |

**Supplementary Table 3. Descriptive statistics for average run-lengths in all conditions and game parts.** Each participant carried out all three parts of the experiment (pre-game, game, and post-game).

| **Condition** | **Experiment part** | **Mean** | **STD** |
| --- | --- | --- | --- |
| Unaware | Pre-Game | 1.269 | 0.128 |
| Unaware | Game | 1.420 | 0.169 |
| Unaware | Post-Game | 1.40 | 0.271 |
| Aware | Pre-Game | 1.330 | 0.190 |
| Aware | Game | 1.423 | 0.178 |
| Aware | Post-Game | 1.419 | 0.227 |
| Control | Pre-Game | 1.273 | 0.192 |
| Control | Game | 1.312 | 0.260 |
| Control | Post-Game | 1.290 | 0.184 |

**Supplementary Table 4. Descriptive statistics for LZC by gender.** Each participant carried out all three parts of the experiment (pre-game, game, and post-game).

| **Gender** | **Condition** | **Experiment part** | **Mean** | **STD** |
| --- | --- | --- | --- | --- |
| Female | Unaware | Pre-Game | 0.947 | 0.027 |
| Female | Unaware | Game | 0.978 | 0.029 |
| Female | Unaware | Post-Game | 0.96 | 0.039 |
| Male | Unaware | Pre-Game | 0.961 | 0.03 |
| Male | Unaware | Game | 0.975 | 0.02 |
| Male | Unaware | Post-Game | 0.956 | 0.033 |
| Female | Aware | Pre-Game | 0.967 | 0.027 |
| Female | Aware | Game | 0.982 | 0.026 |
| Female | Aware | Post-Game | 0.968 | 0.03 |
| Male | Aware | Pre-Game | 0.951 | 0.033 |
| Male | Aware | Game | 0.979 | 0.023 |
| Male | Aware | Post-Game | 0.964 | 0.027 |
| Female | Control | Pre-Game | 0.945 | 0.046 |
| Female | Control | Game | 0.897 | 0.094 |
| Female | Control | Post-Game | 0.954 | 0.025 |
| Male | Control | Pre-Game | 0.936 | 0.052 |
| Male | Control | Game | 0.853 | 0.096 |
| Male | Control | Post-Game | 0.926 | 0.05 |
